# The role of interleukin-1 in perinatal inflammation and its impact on transitional circulation

**DOI:** 10.3389/fped.2023.1130013

**Published:** 2023-03-13

**Authors:** Josephine C. Owen, Steven P. Garrick, Briana M. Peterson, Philip J. Berger, Marcel F. Nold, Arvind Sehgal, Claudia A. Nold-Petry

**Affiliations:** ^1^ Ritchie Centre, Hudson Institute of Medical Research, Melbourne, VIC, Australia; ^2^ Department of Paediatrics, Monash University, Melbourne, VIC, Australia; ^3^ Monash Newborn, Monash Children’s Hospital, Melbourne, VIC, Australia

**Keywords:** perinatal inflammation, transitional circulation, interleukin-1, chorioamnionitis, fetal growth restriction

## Abstract

Preterm birth is defined as delivery at <37 weeks of gestational age (GA) and exposes 15 million infants worldwide to serious early life diseases. Lowering the age of viability to 22 weeks GA entailed provision of intensive care to a greater number of extremely premature infants. Moreover, improved survival, especially at extremes of prematurity, comes with a rising incidence of early life diseases with short- and long-term sequelae. The transition from fetal to neonatal circulation is a substantial and complex physiologic adaptation, which normally happens rapidly and in an orderly sequence. Maternal chorioamnionitis or fetal growth restriction (FGR) are two common causes of preterm birth that are associated with impaired circulatory transition. Among many cytokines contributing to the pathogenesis of chorioamnionitis-related perinatal inflammatory diseases, the potent pro-inflammatory interleukin (IL)-1 has been shown to play a central role. The effects of utero-placental insufficiency-related FGR and in-utero hypoxia may also be mediated, in part, via the inflammatory cascade. In preclinical studies, blocking such inflammation, early and effectively, holds great promise for improving the transition of circulation. In this mini-review, we outline the mechanistic pathways leading to abnormalities in transitional circulation in chorioamnionitis and FGR. In addition, we explore the therapeutic potential of targeting IL-1 and its influence on perinatal transition in the context of chorioamnionitis and FGR.

## 1. Introduction

Chorioamnionitis and fetal growth restriction (FGR) – also referred to as intrauterine growth restriction (IUGR) – are conditions that complicate the course of pregnancy and may predispose infants to morbidity and mortality in early life. Chorioamnionitis involves inflammation of the placenta and fetal membranes (1, 2), whereas FGR is a condition where a fetus fails to reach its genetic growth potential (3). The pathophysiology of both is multifactorial and heterogenous; however, inflammation is a common underlying factor. In a study of 20,091 births (15,710 term and 4,381 preterm), 50.6% of preterm births were linked to placental mal-perfusion, which was associated with FGR, and 27.3% were linked to inflammation/infection (4). Hence, the growing preclinical and clinical evidence for dysregulation of the pro-inflammatory cytokine interleukin (IL)-1 (5–10) in both conditions is the focus of this mini-review.

## 2. IL-1 and its receptors – a brief overview

IL-1 comprises two distinct proteins and master regulators of inflammation, IL-1α and IL-1β. Both require protease processing regulated by inflammasomes to activate their pro-forms (11). One such inflammasome is the nucleotide-binding oligomerization domain-like receptor (NLRP)3 inflammasome. Assembly of the NLRP3 inflammasome is key in the activation of caspase-1, which cleaves pro-IL-1β, allowing for the secretion of its biologically active form, IL-1β (12, 13). Thus, production of IL-1α and IL-1β is controlled by transcription, maturation, and release.

IL-1α and IL-1β are expressed in a wide range of tissues and immune cells and activate pro-inflammatory transcription pathways (14–16) by signaling through the same receptor complex IL-1R1:IL-1R3 (17–19).

The IL-1 receptors contain a cytoplasmic Toll-IL-1-Receptor (TIR) domain that is common to Toll-like receptors (TLRs), which are critical for innate host defense (20), including responses to the intrauterine infections driving preterm delivery (21–23). IL-1 receptors are expressed by a wide variety of cells, resulting in a diverse range of responses upon receptor activation, such as expansion of CD4^+^ T cells (24), increased production of neutrophil chemoattractants (25), and increased permeability of endothelial tissue (26).

Natural counterregulatory mechanisms curtailing IL-1 function comprise IL-1 receptor antagonist (IL-1Ra), which antagonizes the binding of IL-1 to IL-1R1 (27), and the decoy receptor IL-1R2, which transduces no signal upon IL-1α and IL-1β engagement (28).

Blocking IL-1 with its natural adversary IL-1Ra (drug name anakinra) has an excellent safety and efficacy record in inflammatory disease, as established by over two decades of use in adults, children, and infants (29–34). Other trialed strategies of blocking IL-1 [reviewed in (29)] include soluble IL-1 receptor (35), neutralizing IL-1β (36, 37) or IL-1R1-blocking antibodies (38).

## 3. IL-1 and labor onset

In rodent models, IL-1 signaling is not necessary to ensure fertility and initiate labor, as mice deficient in IL-1β (39), caspase-1 (40), or IL-1R1 were fully fertile and delivered at term (41, 42). However, an association between IL-1 and labor has been established in non-human primate models of preterm labor. Pregnant rhesus macaques given an intra-amniotic (i.a.) infusion of IL-1β developed uterine contractions that resulted in preterm labor. In comparison, only 40% of monkeys infused with TNF had uterine contractions, and infusion of IL-6, IL-8 or saline (43) did not result in preterm labor.

In vitro, IL-1 has been suggested to promote labor by increasing calcium concentrations (44) and prostaglandin production (45) in human myometrial cells. Increased prostaglandin abundance has also been observed in the amniotic fluid of women in preterm labor (correlated with IL-1) (46) and in porcine fetal membranes stimulated with IL-1β (47).

Clinical associations between increased IL-1 production and labor onset have also been observed. At late term pregnancy, cervicovaginal fluid abundance of IL-1α and IL-1β peaked 4–14 days prior to spontaneous labor onset, whilst the anti-inflammatory IL-1Ra decreased (48). *IL1B* expression was low in gestational tissues from women not in labor but was present in both maternal and fetal tissues during labor, regardless of GA or intrauterine infection (49). Notably, gene expression data on *IL1B* needs to be interpreted with caution; unless a further activation step triggers IL-1β protein production, *IL1B* mRNA is rapidly degraded.

Even during a healthy pregnancy, the infiltration of leukocytes such as neutrophils, monocytes and macrophages was observed in placental tissue preceding spontaneous labor (50–52). Biopsies from women undergoing cesarean section after the onset of labor revealed that IL-1β was localized to leukocytes in the myometrium, cervix, and fetal membranes (53). Moreover, IL-1β in the amniotic fluid of women at term pregnancy correlated with the degree of leukocytic infiltration in the chorionic membrane (54). In addition to IL-1, pro-inflammatory cytokines IL-6 and IL-8 were rarely found in reproductive tissues pre-labor, but readily found following labor (49, 53, 54). Notably, IL-6 and IL-8 are both induced by IL-1. This indicates that inflammatory processes, especially those driven by IL-1 and originating in infiltrating leukocytes, play a central role in pregnancy and parturition [reviewed in (55)] (Table 1).

## 4. Chorioamnionitis

### 4.1. Clinical association of IL-1 in maternal and fetal chorioamnionitis-affected tissues

**Figure 1:**
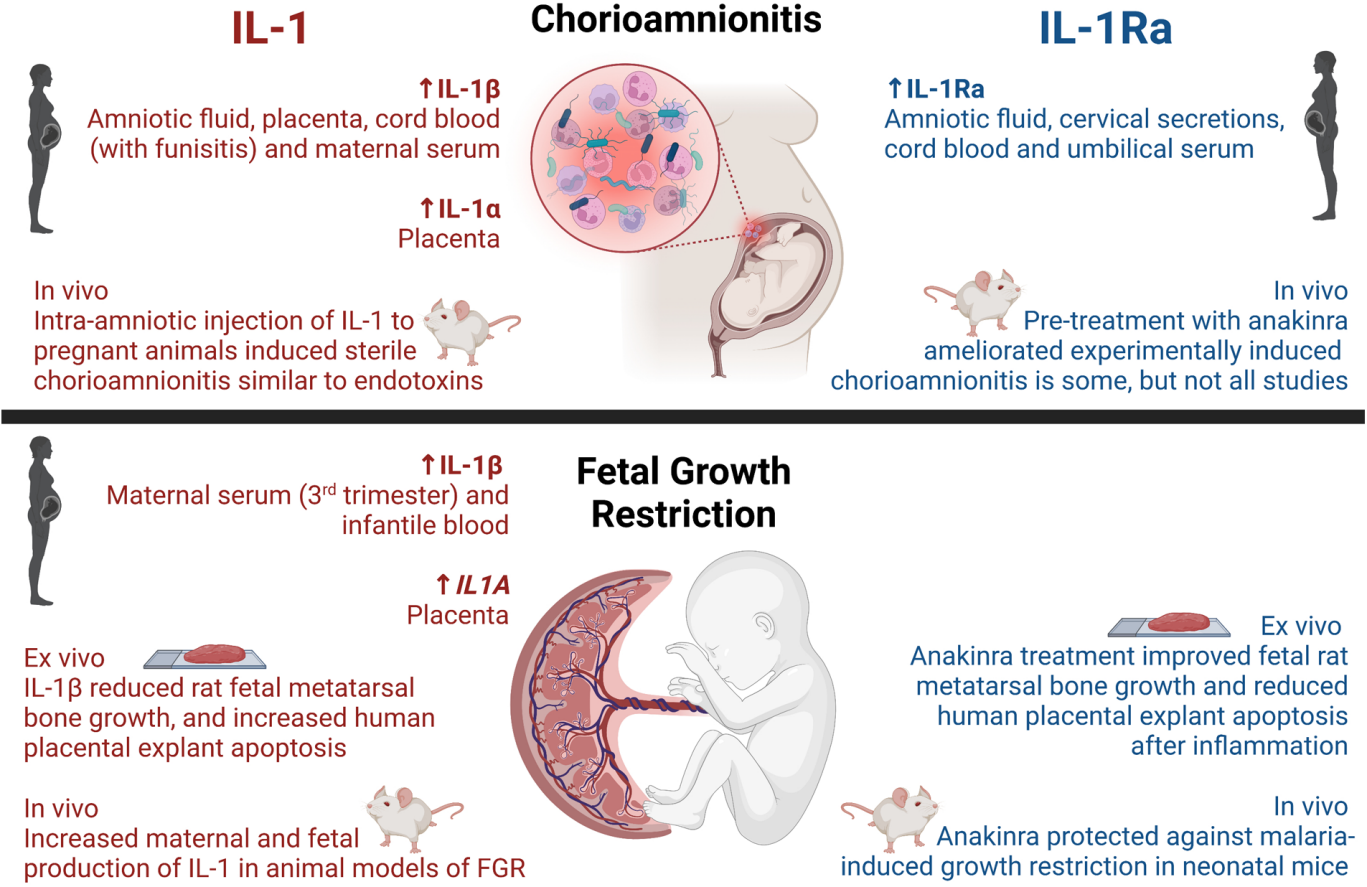
Reported activities of IL-1 and IL-1Ra on chorioamnionitis and fetal growth restriction in humans and disease models. Created with BioRender.com.

In chorioamnionitis, IL-1β abundance was shown to be increased in maternal and fetal tissues (Figure 1 and Table 1), particularly in the amniotic fluid, placenta, maternal blood as well as cord blood in some instances.

**Table 1:** Effects and associated studies of IL-1.

Preclinical evidence	Abundance in humans
**Labor Onset**	
Mice deficient in IL-1β (39), caspase-1 (40), or IL-1R1 (41, 42) are fully fertile and deliver at term. Intra-amniotic IL-1β triggers uterine contractions and preterm labor in a pregnant rhesus macaque model (43). Increased IL-1β abundance correlates with leukocyte infiltration in the chorionic membrane (54) and placental tissue (50–52) preceding spontaneous labour.	IL-1β is elevated in myometrium, cervix and fetal membranes during labor (53). Elevated IL-1α and IL-1β, and reduced IL-1Ra is observed in cervicovaginal fluid 4–14 days prior to spontaneous labor onset (48). *IL1B* expression is increased in fetal and maternal tissues during labor (49). Elevated IL-1Ra in maternal serum correlates with increased risk of preterm birth (56).
**Chorioamnionitis**	
Animal models of chorioamnionitis demonstrate: • Increases in *IL1B* mRNA in rat placenta (57), and sheep chorion-amnion (58, 59). • Increases in IL-1β in maternal serum and amniotic fluid of guinea pigs (60). • Increases in IL-1β in amniotic fluid of rhesus macaques (7). Intrauterine recombinant IL-1β induces sterile chorioamnionitis similar to LPS in a preterm pregnant rhesus macaque model (8). Anakinra (human recombinant IL-1Ra): • Protective against GBS-induced fetal inflammatory response syndrome and neurobehavioral impairment in a rat model of chorioamnionitis (9). • Attenuates lung inflammation in a fetal sheep model of chorioamnionitis (61). • Protective against neutrophil infiltration and increased IL-6 and PGE_2_ abundance in amniotic fluid in a rhesus macaque model of chorioamnionitis (62). • Does not ameliorate LPS-induced inflammation in a sheep model of chorioamnionitis (59). • Does not prevent the increase of pro-inflammatory T cells in fetal spleen from a rhesus macaque model of chorioamnionitis (62). A non-competitive allosteric IL-1Ra (rytvela) reduces IL-1β and CCL2 abundance in amniotic fluid in a sheep model of chorioamnionitis (63).	IL-1β is elevated in amniotic fluid (5, 46, 64–70), placenta (6), and maternal serum (71–73) from chorioamnionitis-affected pregnancies. *IL1B* mRNA expression is increased in maternal serum (74) during chorioamnionitis. Conflicting evidence: reports of increased IL-1 in cord blood from chorioamnionitis-affected pregnancies (75–77) however not in all studies (78–81). May require concurrent funisitis (77). IL-1Ra is increased in amniotic fluid, cervical secretions and cord blood (82–85) from chorioamnionitis-affected pregnancies. Polymorphisms of *IL1RN* are associated with acute deciduitis (86).
**Fetal growth restriction (FGR)**	
Animal models of FGR demonstrate: • Increased IL-1 abundance in murine embryo brain (87). • Increased *Il1* mRNA expression in rat placenta (10), neonatal rat liver (89), and fetal rabbit kidney (90). Growth-restricted offspring in a FGR piglet model demonstrate reduced serum IL-1 (91, 92) and compromised cellular immune responses (88). Treatment of human term placental explants with uric acid crystals induces a pro-inflammatory profile including increased IL-1β abundance, and these effects are IL-1 dependent, and prevented by treatment with anakinra (93). Administration of these uric acid crystals to pregnant rats result in FGR (93). Anakinra ameliorates IL-1β- and TNF-induced suppression of fetal rat metatarsal bone growth (94). Anakinra restores fetal growth in malaria-induced FGR in mice (95).	IL-1β is elevated in maternal serum from third trimester pregnancies affected by placental insufficiency and FGR (96, 97), and in peripheral blood from growth-restricted or -limited infants (98). *IL1A* mRNA expression is increased in placentas from growth-restricted pregnancies (99). Polymorphisms in *IL1A* and *IL1B* are not significantly associated with FGR (100). No significant differences in IL-1 abundance are observed in amniotic fluid and cord blood between growth-restricted and appropriately grown infants (101–103).
**Cardiac Dysfunction**	
IRAK1 deficient mice are resistant to LPS-induced contractile dysfunction (104). Animal models of intrauterine inflammation demonstrate: • Reduced neonatal descending aorta and middle cerebral artery blood flow velocities in rats (105). • Reduced fetal cardiac output with increased cardiac afterload in mice (106). • Impaired cardiac development in neonatal lambs and pigtail macaques (107, 108). • However, the link to IL-1 here is understudied.	Increased ventricular compliance and reduced contractile function are observed in infants exposed to chorioamnionitis (109, 110). Histological chorioamnionitis is associated with: • Higher baseline fetal heart rate and increased periods of low variability (111). • Increased abundance of IL-1β and IL-6 in cord blood, increased heart rate, and decreased blood pressure in the first week post birth (76).
**Pulmonary Inflammation and Maturation**	
Increased lung compliance and improved lung functionality are seen in fetal rabbit and lamb models of chorioamnionitis (112, 113), as well as after intra-amniotic injection with IL-1α (114). In murine models, BPD is precipitated by a rise in pulmonary inflammation, and IL-1β plays a key role in its pathogenesis (115–118).	Chorioamnionitis-exposed infants with respiratory distress exhibit an altered lung surfactant lipidome compared to unexposed infants (119). Chorioamnionitis is associated with increased IL-1β in bronchoalveolar lavage fluid (120, 121) and serum (122) from affected infants.
**Patent Ductus Arteriosus (PDA)**	
No studies identified.	Chorioamnionitis predisposes infants to PDA (123). Late-onset sepsis is associated with a higher rate of unsuccessful DA closure with treatment (124). Large PDA is associated with increased pro-inflammatory (IL-1β, IL-8) and anti-inflammatory (IL-1Ra, IL-10) cytokines (125).

PDA, patent ductus arteriosus; LPS, lipopolysaccharide; GBS, group B streptococcus; PGE_2_, prostaglandin E_2_; CCL2, chemokine (C-C motif) ligand 2; FGR, fetal growth restriction; BPD, bronchopulmonary dysplasia; IRAK1, IL-1 receptor associated kinase-1.

It is well-documented that chorioamnionitis, possibly due to the infiltration of leukocytes into the chorion and amnion (1, 64), is associated with an increased abundance of IL-1 in amniotic fluid (5, 46, 64–70), in addition to preterm labor (46, 70) and preterm pre-labor rupture of membranes (PPROM) (5). Additionally, placental tissues from chorioamnionitis-affected deliveries presented with a seventeen-fold increase in IL-1 abundance compared to healthy pregnancies, with a predisposition towards greater placental IL-1β compared to IL-1α (6). In maternal serum, IL-1β was elevated in preterm histological chorioamnionitis (71), term clinical chorioamnionitis (72) and PPROM complicated by chorioamnionitis (73). *IL1B* mRNA in maternal blood was elevated in women with chorioamnionitis complicated by PPROM (74). There is therefore a strong clinical association between chorioamnionitis and increased abundance of IL-1, which might be related to an increased incidence of PPROM and preterm labor.

This relationship between chorioamnionitis and IL-1 abundance in cord blood is less clear. Increased IL-1β was associated with chorioamnionitis in a select number of studies (75–77), however other studies reported no difference (78–81). This discrepancy may be explained by the concurrent presence of funisitis, an indicator of the fetal inflammatory response, marked by focal aggregation of polymorphonuclear leukocytes at the umbilical cord surface (77).

Additionally, there is now ample evidence from amniotic fluid, cervical secretions, and cord blood (82–85) that IL-1Ra, the natural IL-1 inhibitor, also positively correlates with chorioamnionitis. At first glance, elevations of anti-inflammatory mediators seem counterintuitive in this context; however, increases in the abundance of anti-inflammatory/regulatory mediators are well-recognized as part of the immune system's attempt at regaining homeostasis. Examples include increased IL-1Ra in autoimmune diseases such as rheumatoid arthritis (126) or systemic lupus erythematosus (127). Moreover, as IL-1Ra concentrations increased in maternal serum at 22–24 weeks gestation, so did the risk of preterm birth (56). Polymorphisms in the gene encoding IL-1Ra (*IL1RN*) have been associated with acute deciduitis (86). These findings are consistent with an involvement of IL-1 in chorioamnionitis and preterm labor.

### 4.2. IL-1-driven animal models of chorioamnionitis

Animal studies support the key role of IL-1 during chorioamnionitis (Figure 1 and Table 1). Intraperitoneal (i.p.) injection of lipopolysaccharide (LPS; an endotoxin of Gram-negative bacteria) to pregnant dams increased expression of the gene for IL-1β in both sheep chorion (*IL1B)* (58) and rat placenta (*Il1b*) (57), and additionally increased IL-6 and IL-1β in the maternal serum and amniotic fluid of guinea pigs (60). Moreover, pregnant rhesus macaques injected with live Group B Streptococcus (GBS) into the amniotic cavity or choriodecidual space demonstrated increased abundance of IL-1β and TNF from 13 h post-injection, and concurrently reduced the abundance of prolactin (7). Very high levels of prolactin in amniotic fluid suggest an important role in fetal development, and has been speculated to be involved in the balance of water and electrolytes, yet there is no consensus on the function of prolactin during pregnancy (128).

In addition to chorioamnionitis triggering IL-1 production, i.a. injection of IL-1 was shown to induce sterile chorioamnionitis similar to endotoxins such as LPS: Rhesus macaques were injected with either IL-1β or saline 24 or 72 h before preterm delivery via cesarean section at 80% gestation, i.e., equivalent to 32 weeks GA for a human (8). Monkeys exposed to IL-1β 24 h before cesarean exhibited infiltration of predominantly neutrophils into the decidua parietalis, and these decidual neutrophils produced increased pro-inflammatory cytokines such as TNF and IL-8, and more frequently expressed anti-inflammatory *IDO* (indoleamine 2, 3-dioxygenase) mRNA, than vehicle-treated controls (8). These effects were short-lasting however and by 72-hours post-dose the neutrophil infiltration and cytokine production was lower than at 24 h (8). Data on IL-1 blockade in chorioamnionitis are described in Section 7.

## 5. Fetal growth restriction (FGR)

### 5.1. The impact of FGR on transitional circulation

FGR is intimately related to issues in the cardiopulmonary transition to postnatal life, as placental insufficiency results in chronic deprivation of oxygen and nutrients, which the fetus responds to with adaptations in circulation. These adaptations can be conceptualized as “brain-sparing”, whereby blood flow is increased to the brain, as measured by middle cerebral artery Doppler studies (129, 130), whereas flow to the lower body is reduced. Due to placental vascular bed resistance, the fetal heart is subject to increased afterload, resulting in bilateral ventricular hypertrophy and reduced compliance (131, 132). Moreover, greater coronary artery blood flow, a local response seen in FGR infants, was not associated with improved cardiac function (133). As the severity of FGR increases, fetal cardiac dysfunction and myocardial cell damage increase, and perinatal morbidity worsens (134–136). Dysmorphic pulmonary vascular growth increases right to left ductal shunting, further contributing to cardiac dysfunction (137). Despite extensive research into FGR and the transitional circulation, little is known of the role of inflammation and IL-1 in this relationship.

### 5.2. Clinical association of IL-1 with FGR-affected maternal and infantile tissues

Knowledge on the clinical association between FGR and IL-1 in maternal and infantile samples is limited (Figure 1 and Table 1). Maternal serum abundance of IL-1β was elevated in third trimester pregnancies affected by placental insufficiency and FGR, compared to uncomplicated pregnancies (96, 97). Moreover, on day 14 of neonatal life, IL-1β was increased in peripheral blood from growth-restricted or -limited infants (98), and placentas from growth-restricted pregnancies exhibited higher *IL1A* mRNA when compared to healthy pregnancies (99). Polymorphisms in *IL1A*, namely −889C/T and +4,845G/T alleles, were associated with altered transcriptional activity and aberrant production of IL-1α. Females carrying these alleles had an elevated risk of preterm birth, and bearing of low birthweight infants, however no significant difference in the risk of FGR was confirmed (100). These findings suggest that aberrant production of IL-1α may increase the risk of preterm birth, and thus low birthweight, but were not directly associated with FGR. No associations between polymorphisms in *IL1B* and the risk of preterm birth, low birthweight, nor FGR were found (100). When examining amniotic fluid and cord blood, no differences in IL-1 abundance were detected between growth-restricted and appropriately grown infants (101–103).

### 5.3. Dysregulation of IL-1 in animal models of FGR

FGR has been linked to dysregulated IL-1 and related pathways in vivo (Figure 1 and Table 1). Many animal models of FGR have been used to investigate IL-1, including calorie-restriction (89), uteroplacental ligation (90), maternal ethanol exposure (87), bariatric surgery (10) and administration of uric acid (93). These studies demonstrated increased maternal and fetal protein production and gene expression of IL-1α and IL-1β in FGR-affected pregnancies compared to uncomplicated pregnancies.

In FGR piglets, cellular immune responses were compromised and contributed to an increased incidence of sepsis in the postnatal period (88). Piglets born FGR were associated with a lower serum immunoglobulin A and IL-1 when compared to control offspring (91, 92), potentially via increased expression of *HSP70* (heat shock protein 70) (91, 138). HSP70 is a stress-response protein which can be induced by hypoxia-ischaemia (139), and thus may be increased in FGR, as found in one study examining piglet intestines at birth (140). HSP70 is also a danger signal to the innate immune system (141), and if overexpressed, can inhibit cytokine expression (142). Thus, HSP70 overproduction secondary to FGR may compromise cellular immune responses, including IL-1 expression (91).

## 6. Neonatal outcomes

Perinatal inflammation is inversely related to GA and birthweight, and predisposes infants to cardiopulmonary dysfunction and morbidity, as well as patent ductus arteriosus.

### 6.1. Cardiac dysfunction

Exposure to IL-1β has been linked to cardiac depression. IL-1β depressed rat cardiac myocyte contractile function in vitro (143, 144), and isolated hearts from IL-1 receptor associated kinase-1 (IRAK1) deficient mice were resistant to LPS-induced contractile dysfunction ex vivo (104).

Whilst a causal relationship between IL-1 in the transitional circulation and cardiac dysfunction has not yet been established, excessive inflammation in utero, to which IL-1 signaling contributes, induced cardiac dysfunction in neonatal rodents and sheep. The intracervical injection of LPS to pregnant rats on embryonic day (E)15 and subsequent serial transabdominal echocardiogram performed on fetuses in utero revealed a blunted increase in gestation related aortic blood flow velocity (BFC), and a decreased middle cerebral artery BFC compared to the vehicle controls (105). Another study administered LPS i.p. to pregnant mice on E14-15 and after 6 h investigated fetal cardiac dysfunction and inflammatory changes in the placenta. LPS exposed animals revealed increased fetal cardiac afterload, reduced fetal cardiac output, and increased placental expression of *Il1a*, *Il6* and *Tnf* (106).

An association between intrauterine inflammation and long-term vulnerability to cardiac disease is beginning to be explored. Lambs exposed to i.a. injection of LPS 48 h before delivery exhibited impaired cardiomyocyte growth, increased collagen deposition and remodeling of the left ventricular myocardium, when compared to saline-treated controls. Affected lambs also demonstrated increased expression of genes related to cardiac metabolism and calcium handling, however expression of *IL1B* was not significantly increased (107). In non-human primates, cardiac tissue exposed to intrauterine infection identified reduced gene expression of pathways involved in cardiac morphogenesis and vasculogenesis (108). Abundance of IL-6 and IL-8 in cardiac tissue was increased in intrauterine infection, however IL-1 and TNF abundance was not significantly different between groups (108). These studies highlight the long-term implications of intrauterine inflammation on heart disease, beyond the transition to extrauterine life.

In humans with sepsis, circulating depressing factors, such as IL-1β, are speculated to induce myocardial dysfunction (145), and cardiac dysfunction after inflammation is further evidenced in human fetuses exposed to pre-labor rupture of membranes (PROM) or i.a. infections. Affected infants had increased ventricular compliance and reduced contractile function (109, 110). Cardiotocography during PROM also showed that fetuses exposed to histological chorioamnionitis exhibited a higher baseline heart rate and increased periods of low variability (111). Histological chorioamnionitis has been correlated with increased cord blood IL-1β and IL-6, increased heart rate and decreased blood pressure in the first week after birth (76).

Therefore, inflammation in the transitional circulation, as occurring in chorioamnionitis, precipitates cardiac dysfunction, and is speculated to be mediated in part by IL-1 (Table 1), however this needs to be confirmed in future studies.

### 6.2. Pulmonary inflammation and maturation

In addition to cardiac complications, chorioamnionitis and increased IL-1 were associated with pulmonary complications (Table 1). Early life inflammation had an initial maturing effect which increased surfactant production from type-2 alveolar epithelial cells. This increased surfactant production increased lung compliance and improved lung functionality, in experimentally-induced chorioamnionitis (112, 113) or i.a. IL-1α injection (114). Clinically, there is little research on the relationship between chorioamnionitis and surfactant production. However, it was reported that infants with respiratory distress from pregnancies complicated by chorioamnionitis presented with an altered lung surfactant lipidome compared to those without chorioamnionitis (119).

Despite the initial maturing effect of chorioamnionitis on the immature lung, the longer-term outcomes are often poor. Chronic pulmonary inflammation in mice, induced with antenatal inflammation and postnatal hyperoxia, disrupted alveolarization and vasculogenesis, manifesting a lung disease known as bronchopulmonary dysplasia (BPD). This BPD phenotype was precipitated by a rise in pulmonary inflammation, to which IL-1β was established as a key pathogenic factor (115–118, 146). In humans, infants from pregnancies complicated by chorioamnionitis have elevated IL-1β in bronchoalveolar lavage fluid (120, 121) and serum (122). Thus, somewhat paradoxically, chorioamnionitis was associated with a reduced incidence of early respiratory distress (147), but an increased incidence of BPD (148).

### 6.3. Patent ductus arteriosus (PDA)

PDA describes a persistent opening between the aorta and pulmonary artery after birth, affecting up to 55% of infants ≤28 weeks' GA (149) and 31% of infants <1,500 g (150). Patency and closure of the ductus arteriosus (DA) is a complex area of study [as reviewed in (151)]. During early fetal development nitric oxide (NO) is the primary mediator responsible for maintaining patency (152, 153). Closer to term, this role is filled by prostaglandin E_2_ (PGE_2_) (152, 153). Importantly IL-1β is a potent inducer of PGE_2_ expression (154), yet the relationship between IL-1 and PDA remains understudied (Table 1). Infection and inflammation increase the risk of PDA, and is associated with increased cyclooxygenase-1 (155) and 6-keoprostaglandin F1α (156).

After birth, multiple factors contribute to the closure of the DA, a process that is ultimately achieved by smooth muscle constriction (157). Key triggers for this muscular contraction likely include a drop in circulating PGE_2_ (158, 159), and increased calcium activity after acute oxygenation (as is seen in preterm infants with ventilation) (160–162).

Systematic review and meta-analysis of 23 studies, including over 17,708 infants, revealed that chorioamnionitis predisposed infants to PDA (123). Further, another clinical study found that late-onset sepsis (i.e., sepsis occurring later than ∼72 h of life) was associated with a higher rate of unsuccessful closure after treatment with concomitant diuretic and oral paracetamol treatment (124). Accordingly, inflammation is likely to play an important role in persistence of the DA. There is very little research on the relationship between IL-1 and PDA. However, echocardiography and plasma samples taken on day 3 of life in 53 infants, with a GA at birth below 28 completed weeks, revealed an association between large PDA (>1.5 mm) and increased pro-inflammatory (IL-1β, IL-8) and anti-inflammatory (IL-1Ra, IL-10) cytokines (125).

## 7. Blockade of IL-1 in chorioamnionitis

Considering the evidence linking chorioamnionitis with maternal and neonatal morbidity, and the negative effects of excessive inflammation and IL-1 production, anakinra has been investigated in a variety of intrauterine inflammation models (Figure 1 and Table 1).

Pregnant rats that received GBS i.p. at E19, and subsequently received three antenatal i.p. doses of anakinra, exhibited improved gestational weight, reduced IL-1β titer in placentae, maternal and fetal sera, and improved neonatal neurobehavioral outcomes when compared with rats exposed to GBS only (9).

Fetal sheep were exposed to i.a. injections of LPS with or without prior i.a. injection of recombinant human IL-1Ra. IL-1Ra pre-treatment decreased LPS-induced inflammation, as assessed by decreased lung neutrophil and monocyte influx, and decreased lung *IL6* and *IL1B* levels, as well as decreased plasma IL-8. Blockade of IL-1 signaling in the amniotic compartment therefore inhibited fetal inflammation in response to chorioamnionitis (61).

Administration of either IL-1α or LPS i.a. to pregnant sheep resulted in placental inflammation, increased *IL1B*, *IL6* and *IL8* mRNA and IL-8 protein abundance and infiltration of inflammatory cells into the chorio-amnion (59). However, pretreatment with anakinra did not ameliorate the LPS-induced inflammation; most notably C-C Motif Chemokine Ligand 2 (CCL2)-expressing cells in the chorio-amnion were unchanged (59). In a separate study, i.a. LPS injection to pregnant sheep increased amniotic fluid CCL2 after 24 h (63). After fetal intravenous administration of rytvela, a non-competitive allosteric IL-1Ra, amniotic fluid CCL2 was significantly lower compared to controls (63). The differences in outcomes between these studies could be related to dosing or timing of LPS administration or the mechanism of IL-1 blockade.

Treatment of pregnant rhesus macaques with anakinra prior to i.a. administration of LPS prevented increased neutrophil infiltration and increased IL-6 and PGE_2_ abundance in the amniotic fluid, as compared to LPS-only controls (163). In a separate study using the same protocol, anakinra did not prevent the increase in pro-inflammatory T cells and decreased anti-inflammatory regulatory T cells, in the spleen of LPS-exposed fetuses (62).

IL-1R blockade shows promise as a potential therapeutic to reduce intrauterine inflammation and neonatal morbidity as seen in chorioamnionitis. However, more research is needed considering the limited literature.

## 8. Blockade of IL-1 in FGR

FGR is a multifactorial disease, to which inflammation contributes as described. Thus, one could speculate that blocking IL-1 could hold promise as a treatment to improve the outcome or even prevent FGR. However, blockade of IL-1 in preclinical models of FGR remains relatively understudied (Figure 1 and Table 1).

To our knowledge, there are only two ex vivo studies investigating IL-1R blockade in FGR. The first study exposed fetal metatarsal bones from Sprague Dawley rats to IL-1β and TNF, leading to reduced bone growth, which was improved by anakinra in a dose-dependent manner (94). The second study induced apoptosis via uric acid or IL-1β in human placental explants, which could be prevented by caspase-1 inhibition or anakinra treatment (93).

Interestingly, malaria infection during pregnancy led to FGR in infants, which was paralleled by placental activation of the NLRP3 inflammasome and increased *IL1B* expression (95). Antenatal exposure of pregnant mice to a Plasmodium parasite followed by a 5-day therapeutic treatment with anakinra commenced within 24 h after infection, restored fetal growth and reduced fetal resorption (95). Whilst this provides preliminary evidence for treating malaria-induced FGR with IL-1 blockade, further studies are needed to determine whether anakinra protects against other FGR pathologies, including placental mal-perfusion.

## 9. Conclusion

In normal pregnancies, IL-1 contributes to normal parturition and birth. However, in inflammation, e.g., in chorioamnionitis, IL-1 is often increased, and associated with preterm labor. Exposure of the fetus to increased IL-1 also contributes to postnatal inflammation, which can negatively affect the neonatal heart (resulting in myocardial depression) and lungs (increased risk of BPD and PDA). Therapeutic or prophylactic blockade of IL-1 signaling pathways in preclinical models of chorioamnionitis have shown to reduce intrauterine inflammation and improve fetal outcomes. The evidence on IL-1 blockade as a treatment for FGR is preliminary, but opens the field for further studies. Overall, there is good evidence to support the concept of IL-1 blockade for treating perinatal inflammation and to improve transitional circulation.
